# Learning continuous and data-driven molecular descriptors by translating equivalent chemical representations†
†Electronic supplementary information (ESI) available: Detailed information regarding the final model architecture, hyperparameter grid, results and computation time. See DOI: 10.1039/c8sc04175j


**DOI:** 10.1039/c8sc04175j

**Published:** 2018-11-19

**Authors:** Robin Winter, Floriane Montanari, Frank Noé, Djork-Arné Clevert

**Affiliations:** a Department of Bioinformatics , Bayer AG , Berlin , Germany . Email: robin.winter@bayer.com; b Department of Mathematics and Computer Science , Freie Universität Berlin , Berlin , Germany

## Abstract

There has been a recent surge of interest in using machine learning across chemical space in order to predict properties of molecules or design molecules and materials with the desired properties. Most of this work relies on defining clever feature representations, in which the chemical graph structure is encoded in a uniform way such that predictions across chemical space can be made. In this work, we propose to exploit the powerful ability of deep neural networks to learn a feature representation from low-level encodings of a huge corpus of chemical structures. Our model borrows ideas from neural machine translation: it translates between two semantically equivalent but syntactically different representations of molecular structures, compressing the meaningful information both representations have in common in a low-dimensional representation vector. Once the model is trained, this representation can be extracted for any new molecule and utilized as a descriptor. In fair benchmarks with respect to various human-engineered molecular fingerprints and graph-convolution models, our method shows competitive performance in modelling quantitative structure–activity relationships in all analysed datasets. Additionally, we show that our descriptor significantly outperforms all baseline molecular fingerprints in two ligand-based virtual screening tasks. Overall, our descriptors show the most consistent performances in all experiments. The continuity of the descriptor space and the existence of the decoder that permits deducing a chemical structure from an embedding vector allow for exploration of the space and open up new opportunities for compound optimization and idea generation.

## Abstract 2

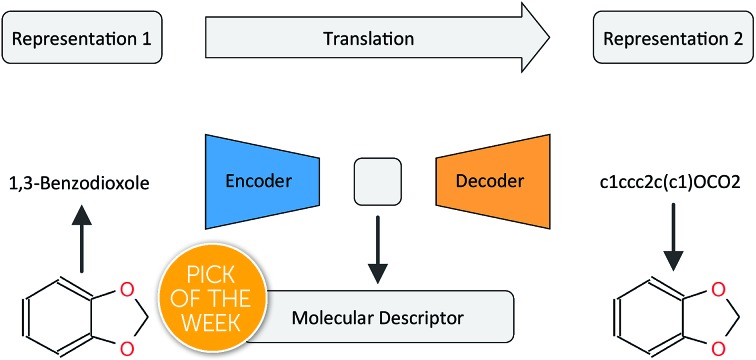
Translation between semantically equivalent but syntactically different line notations of molecular structures compresses meaningful information into a continuous molecular descriptor.

## 1 Introduction

Molecular descriptors play a crucial role in chemoinformatics, since they allow representing chemical information of actual molecules in a computer-interpretable vector of numbers.^1^ While chemical information can be represented by experimental measurements such as physico-chemical property measurements,^2^ a lot of work has been done to derive molecular descriptors from a symbolic representation of a molecule. A widely used concept to generate such theoretical molecular descriptors is *molecular fingerprints*. Molecular fingerprints encode structural or functional features of molecules in a bit string format and are commonly used for tasks like virtual screening, similarity searching and clustering.^3^–^5^ In particular, circular fingerprints like the *extended-connectivity fingerprints* (ECFPs) were introduced to model quantitative structure–activity relationships (QSAR) for biological endpoints by way of classical machine learning approaches as well as for ligand-based virtual screening (VS).^6^,^7^

Recent advances in the field of Deep Neural Networks (DNNs)^8^,^9^ also showed an impact in chemoinformatics-related tasks such as molecular property and activity prediction.^10^–^12^ The proposed DNNs have in common that they use pre-extracted molecular descriptors (mostly ECFPs) as input features. This, however, contradicts the fundamental idea of representation learning: DNNs should learn a suitable representation of the data from a simple but complete featurization, rather than relying on sophisticated human-engineered representations.^9^,^13^

Following these considerations, work was also done to apply DNNs directly on supposedly more complete and lower-level representations of a molecule such as the molecular graph^14^ or the sequential SMILES (Simplified Molecular Input Line Entry Specification) representation.^15^–^17^ By training a DNN directly on a comprehensive and low-level representation, it can automatically learn to extract useful descriptors best suited for the specific task it is trained on, resulting in a specific descriptor set for a given dataset. The downside, however, directly follows from this property. Since features have to be learned from scratch for every new dataset, these methods are prone to overfitting if trained on limited data. This is an issue when it comes to bioactivity data, due to the relatively high cost of generating a data point.^11^,^18^,^19^

Recently, work was also done to learn molecular descriptors in an unsupervised and data-driven way. Gómez-Bombarelli *et al.* proposed a *variational autoencoder*^20^ to convert the discrete SMILES representation of a molecule to and from a multidimensional continuous representation.^21^ Although their main purpose was to build a framework for *de novo* molecular design, the authors showed that the resulting representations could also be used as descriptors for a down-stream classification task. Xu *et al.* proposed a related unsupervised approach based on *sequence to sequence learning*.^22^,^23^

Both studies use an *autoencoder*^24^ methodology applied on the SMILES representation. An autoencoder comprises two neural networks, an encoder and a decoder. The encoder network transforms the input, here a SMILES sequence of variable length with discrete values, to a fixed size continuous representation (*latent representation*). The decoder network takes the latent representation as the input and aims at transforming it back to the input sequence. The whole autoencoder network is trained on minimizing the mean reconstruction error on a single-character level for each input sequence. By introducing an *information bottleneck* between the encoder and the decoder, the network is forced to compress the essential information of the input, so that the decoder still makes as few errors as possible in the reconstruction. If the trained autoencoder is able to encode all the necessary information of a given molecular representation to accurately reconstruct the original molecular representation, Xu *et al.* argue that it may also capture more general chemical information about the molecule and could be used as a molecular descriptor. However, training an autoencoder on reconstructing a sequence which represents a molecule bears the risk that the network solely focuses on syntactic features and repetitive patterns of this sequence, neglecting its semantics and failing to encode higher-level concepts such as molecular properties.

**Fig. 1:**
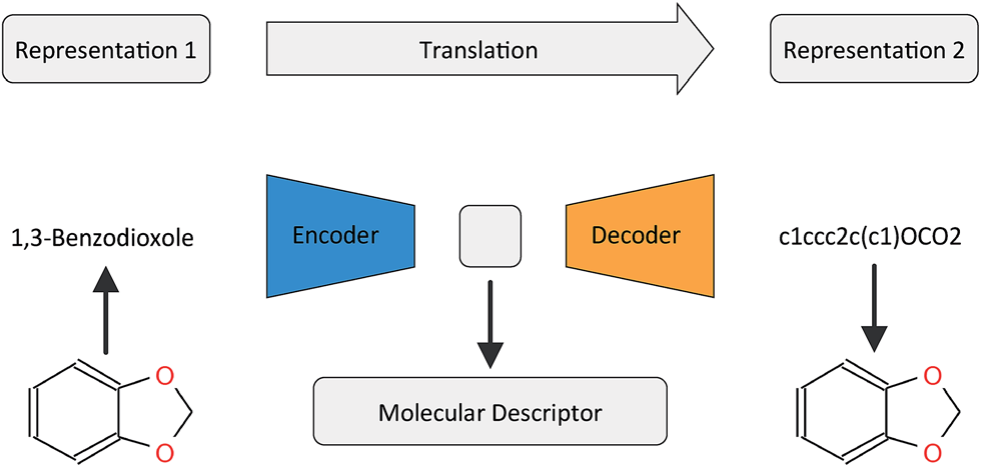
General architecture of the translation model using the example of translating between the IUPAC and SMILES representations of 1,3-benzodioxole.

In this work, we want to address this issue by proposing a method that is based on a translation rather than a reconstruction methodology (see Fig. 1). Similar to a human translating a sentence from one language to another by first reading the whole sentence to get a general understanding before starting translation, a so-called Neural Machine Translation (NMT)^23^ model first reads the whole input sequence and encodes it into an intermediate continuous vector representation (latent representation) which is then used by the decoder to emit a respective translation. This latent representation can be thought of as the model's “understanding” of the input sequence's “meaning”, incorporating all the semantic information shared by the input and output sequences. Here, we want to exploit this translation methodology to extract the “meaning” of a molecular representation like an InChI (International Chemical Identifier)^25^ by translating it to another syntactically different one, *e.g.* SMILES. Since the decoder uses the encoded latent representation to generate a semantically equivalent but syntactically different representation, the network does not benefit from encoding unnecessary information about the input sequence. However, the decoder can only succeed in generating the right translation for a given molecular representation if the encoder compresses a comprehensive description of the chemical structure in the latent representation.

By training the translation model in a data-driven way on a large set of chemical structures, we propose a model that can extract the information contained in a comprehensive but discrete and variable-sized molecular representation (*e.g.* SMILES) and transform it into a continuous and fixed-sized representation. Once trained, the resulting model can be used to extract meaningful molecular descriptors for query structures without the need for retraining or including labels. To analyse the quality of the resulting molecular descriptors, we perform a variety of experiments on predictive QSAR and virtual screening tasks. Finally, we show that it is possible to navigate smoothly in this new continuous chemical descriptor space by modifying slightly the molecular representation of an existing compound in a given direction and using the decoder to obtain new chemical structures.

## 2 Methods

### 2.1 Molecular representations

While translation could be performed between arbitrary molecular representations, in this work we focus on the sequence-based SMILES and InChI representations.

The InChI notation represents molecular structures as a sequence of characters divided into layers and sub-layers providing different types of information such as the chemical formula, bonds and charges.

The SMILES notation also represents molecular structures as a sequence of characters. In contrast to the InChI notation, however, a SMILES is not divided into different information layers but encodes the whole molecular structure in one sequence of characters including identifiers for atoms as well as identifiers denoting topological features like bonds, rings and branches. Since a molecule can typically be represented by many of equally valid SMILES, various algorithms have been developed to guarantee the uniqueness of a SMILES notation for a molecule. In this work we use the library RDKit^26^ to generate such canonical SMILES.

Table 1 visualizes how the different notations differ in their syntax while representing the same molecule. Although both SMILES and canonical SMILES share the same identifiers and general syntax, the two sequences, coming from different algorithms, are not identical. Hence, not only could translation be performed between InChI and canonical SMILES, but also between any SMILES representation of a molecule and its canonical version. We utilized the SMILES enumeration procedure proposed by E. Bjerrum to generate a random SMILES variant for a given molecule.^16^ In order to be invariant to the SMILES representation at inference time, we also used the canonical SMILES as the input half of the time.

**Table 1:** Different sequence-based molecular representations for the example 1,3-benzodioxole

Graph	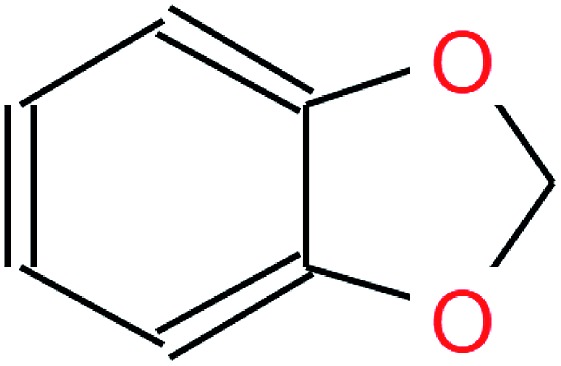
IUPAC	1,3-Benzodioxole
SMILES	c1ccc2c(c1)OCO2
Canonical SMILES	c2ccc1OCOc1c2
InChI	InChI = 1S/C7H6O2/c1-2-4-7-6(3-1)8-5-9-7/h1-4H,5H2

In order to use the aforementioned sequence-based molecular representations as the input and output of the translation model, we tokenized the sequences and encoded them in a one-hot vector representation. By defining a lookup table T for the *N* tokens in sequence representations (*e.g.* T_2_ = C, T_5_ = Br), a one-hot representation of token T_*i*_ is defined by an *N*-dimensional vector with a one in the *i*-th entry and zeros elsewhere. We defined different lookup tables for both SMILES and InChI representations, mostly tokenizing the sequences on a character level except for “Cl”, “Br” and “InChI = 1S/”. We tokenized 38 and 28 unique characters for SMILES and InChI sequences, respectively.

### 2.2 Translation model

Fig. 1 depicts the general concept of the model for an example of translating from the IUPAC representation of a given molecule to its SMILES representation. For the encoder network, we tried both convolutional neural network (CNN) and recurrent neural network (RNN) architectures of different size and depth followed by a fully connected layer that maps the output of the CNN or the concatenated cell states of the RNN to the latent space, respectively (see the ESI† for an introduction to the basic concepts of these different neural network architectures). The decoder network consists of an RNN, whose cell states are initialized by an individual fully connected layer for each layer in the RNN, taking the latent space as the input.

To further encourage the model to learn a meaningful representation of a molecule, we extend the translation model by an additional classification model for certain molecular properties. Similar to the method proposed by Gómez-Bombarelli *et al.*, this classification model takes the latent representation of the translation model as the input and predicts certain molecular properties which can be directly deduced from the molecular structure. We fixed the classification model as a 3-layer fully connected neural network, mapping the latent space to the molecular property vector.

The output of the decoder network's RNN is a sequence of probability distributions over the different possible characters defined in the respective lookup table. The complete model is trained on minimizing the cross-entropy between these probability distributions and the one-hot encoded correct characters in the target sequence as well as minimizing the mean squared error in predicting the molecular properties (classifier network). For the decoder RNN we utilized *teacher forcing*^27^ during training and a left-to-right beam search^23^ during inference.

We monitored the translation accuracy of the model on a single-character level, by comparing the correct character in the target sequence with the most probable character in the decoder RNN's output at each position. To select the best combination of translation task and architecture, we used the predictive performance of machine learning models built on two QSAR datasets using the respective latent representations as descriptors. This ensures that the translation model not only works well at translating (high single-character accuracy) but is also well suited to extract meaningful molecular descriptors from the input sequence (good performance of a simple QSAR model build on the embedding).

### 2.3 Datasets and preprocessing

The translation model was pretrained on a large dataset composed of molecular structures from the ZINC15 (ref. 28) and PubChem^29^ databases. Both databases were merged, the duplicates removed and filtered with RDKit using the following criteria: only organic molecules, molecular weight between 12 and 600, more than 3 heavy atoms and a partition coefficient log *P* between –7 and 5. Additionally, we removed the stereochemistry, stripped the salts and only kept the largest fragments. For each molecule, nine molecular properties were extracted: log *P*, the maximal and minimal partial charge, the number of valence electrons, the number of hydrogen bond donors and acceptors, Balaban's *J* value,^30^ the molar refractivity and the topological polar surface area. Molecules which could not be processed by RDKit were removed. After applying this preprocessing procedure the resulting dataset consisted of approximately 72 million compounds.

For the evaluation of the molecular descriptors extracted by the final translation model, we performed eight QSAR and two VS experiments. The QSAR datasets (see Table 2) were taken from various sources and were preprocessed in the same way as the pretraining dataset. Two of the datasets (Ames mutagenicity and lipophilicity) were used to validate the different translation models' architectures. The remaining eight datasets were solely used for evaluating the final model.

**Table 2:** Ten different QSAR datasets used for benchmarking our molecular descriptor. The final number of compounds in each task after preprocessing is mentioned

Dataset	Acronym	Task	Split	Number of compounds	Reference
Ames mutagenicity	ames	Classification	Validation	6130	33
HERG inhibition	herg	Classification	Test	3440	34
Blood–brain barrier penetration	bbbp	Classification	Test	1879	35
β-Secretase 1 inhibition	bace	Classification	Test	1483	36
Toxicity in honeybees	beet	Classification	Test	188	37
Epidermal growth factor inhibition	egfr	Regression	Test	4451	38
Plasmodium falciparum inhibition	plasmo	Regression	Test	3999	39
Lipophilicity	lipo	Regression	Validation	3817	40
Aqueous solubility	esol	Regression	Test	1056	41
Melting point	melt	Regression	Test	184	42

The VS experiments were performed on 40 targets of the Directory of Useful Decoys (DUD) and 17 targets of the Maximum Unbiased Validation (MUV) dataset.^31^,^32^

All compounds of the evaluation datasets were removed from the pretraining dataset.

### 2.4 Evaluation and baseline

Our new molecular descriptors were benchmarked against state-of-the-art descriptors in QSAR and VS experiments.

For modelling structure–activity relationships, we compare three different approaches: classical machine learning models applied on our descriptors and on circular fingerprints of different radii and folding as implemented in RDKit (see details in the ESI†) as well as an end-to-end molecular graph convolution method as implemented in DeepChem.^43^ The first two methods require selecting the learning algorithm to plug on top of the molecular representation. For this, we used Random Forest (RF),^44^ support vector machine (SVM) with an RBF kernel^45^ and Gradient Boosting (GB)^46^ as implemented in scikit-learn.^47^ A preliminary check on the two validation tasks showed that SVM was the method that worked best in combination with our descriptors and was therefore the only method applied to all other QSAR datasets for our descriptors. Our descriptors were standardized to zero mean and unit variance for each task individually. We performed an extensive hyperparameter optimization in a nested cross-validation (CV) fashion to select the best set of descriptor, model and hyperparameters for each task (see the ESI† for the detailed hyperparameter grid for each model).

The graph convolution models were trained directly on the different QSAR datasets. Hyperparameters such as learning rate and filter size were optimized in a cross-validation (see the ESI† for the detailed architecture and hyperparameter grid).

Each dataset was split in two different ways for the validation. The random CV corresponds to five random splits while the cluster CV corresponds to five clusters obtained by *K*-means clustering with *K* = 5 on MACCS fingerprints.^48^

To select the best performing combinations, we specifically looked at the coefficient of determination (*r*^2^) and the area under the receiver operating characteristic curve (ROC AUC) for the regression and classification tasks, respectively.

For the ligand-based virtual screening experiments, we followed the benchmarking protocol proposed by Riniker *et al.*^49^ For each target in both VS databases, five active compounds were picked randomly and the remaining compounds were ranked according to their similarity to the active set as measured by a similarity metric in the respective descriptor space. The process was repeated 50 times for each dataset, each time selecting a new random set of active and decoy compounds. We compared the performance of our descriptors with the 14 molecular fingerprints provided in the benchmark protocol (see the ESI†). The similarity in the discrete baseline fingerprint space was calculated using the Tanimoto similarity. For our continuous descriptors (per-target standardized to zero mean and unit variance) we used cosine similarity. The resulting ranking of the compounds is evaluated by calculating the mean ROC-AUC over the 50 repetitions for each target. Additionally, a Wilcoxon signed-rank test^50^ is performed to analyse the statistical significance of the differences in the mean ranks of our descriptor to the baseline descriptors.

## 3 Results and discussion

Our translation model is a data-driven method for generating meaningful compound representations by forcing translation of all necessary information between two sequence-based representations of a molecule into a low dimensional continuous embedding (latent space). Since sequence-based representations of molecules such as SMILES or InChI are easily obtained from cheminformatics packages, the pretraining of the model can be performed on a vast chemical space (here, around 72 million compounds were used). Once the pretraining is finalized, the translation model can be used to encode compounds into the embedding or to decode embeddings into compounds. The obtained compound embedding can be utilized as a new continuous and reversible molecular descriptor that can be used to evaluate similarity in chemical space or train machine learning models to predict properties and biological activities.

### 3.1 Pretraining

**Fig. 2:**
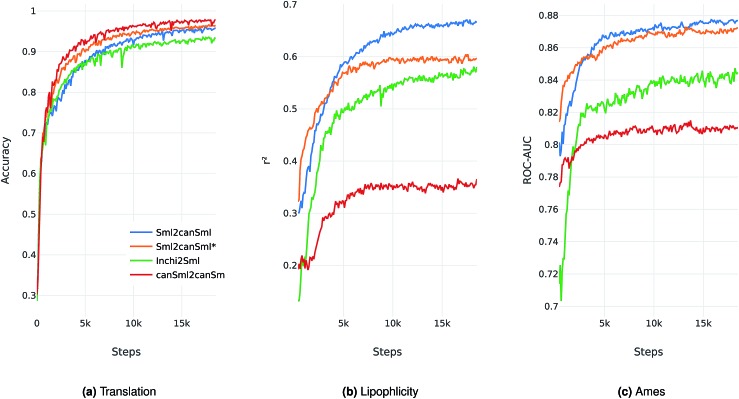
Performance of the best model on four different translation tasks during the first 20 000 training steps. The Sml2canSml* run was trained without the additional classification task of molecular properties. (a) Translation accuracy. (b) Mean performance on the lipophilicity regression task. (c) Mean performance on the Ames classification task. For (b) and (c), the translation model at the respective step was utilized to extract the molecular descriptors fed into an SVM to model both tasks.

We evaluate the different network architectures of the translation model (see Fig. 1) in terms of performance of the extracted descriptors for the two validation tasks. Fig. 2 shows both translation accuracy and predictive performance on the validation sets during the first 20 000 training steps. We show the best performing model for both translation tasks (SMILES to canonical SMILES and InChI to canonical SMILES) as well as the best model for the regular canonical SMILES autoencoding task.

Generally, as the models get better at translating the input to the output sequence, the predictive performance of an SVM based on the latent representation also improves. Since the translation model is trained on producing the correct translated sequence for a given input sequence, it is forced to store all important information necessary to do this translation in the bottleneck of the network: the latent representation (see Fig. 1). The more the information of a molecule encoded in the latent representation, the better it is suited as a molecular descriptor to predict certain properties of this molecule. Hence, the prediction performance on QSAR tasks increases.

The overall best performance was achieved with a translation model based on an RNN architecture for the encoder network that was trained on translating from a SMILES representation to its canonical version (see the ESI† for the detailed network architecture). The model based on the InChI to canonical SMILES translation is also able to accurately translate between the two representations. Its intermediate latent representation, however, is not as well suited for training an SVM on the validation task.

We also tried to train models on translating from canonical SMILES to InChI representations. These models, however, failed (in contrast to the opposite task) to learn anything. This is probably due to the higher complexity of the InChI format (including counting and arithmetic as already discussed by Gómez-Bombarelli *et al.*), making the generation of a correct InChI string for a given molecule a difficult task to learn.

In order to assess the impact of the additional classification task of molecular properties, Fig. 2 also shows the performance of the best model without this additional task during training. Since this model solely focuses on translating, it reaches better translation accuracies faster. However, this difference seems to diminish as training time increases. The additional classification task seems to have a clear positive impact on the predictive performance of the lipophilicity task, while resulting in a small improvement on the Ames mutagenicity task. The improvement on the lipophilicity task is probably mainly due to its correlation with molecular properties (such as the partition coefficient log *P*) that were included in the classification task.

All models based on translating between two different molecular representations show a clear improvement over models trained on reconstructing the same input sequence. Interestingly, translating between two molecular representations seems harder to learn than reconstructing the same input representation (see Fig. 2a). This can be explained by the fact that the translation models cannot simply store sequence-based features or patterns in the latent space, but have to learn to extract the information that both the input and output sequences have in common: the molecule they are both representing. These findings imply that, indeed, the translation task encourages the model to encode more relevant information of the molecule in the latent space, resulting in a potentially powerful molecular descriptor.

### 3.2 QSAR modelling

**Fig. 3:**
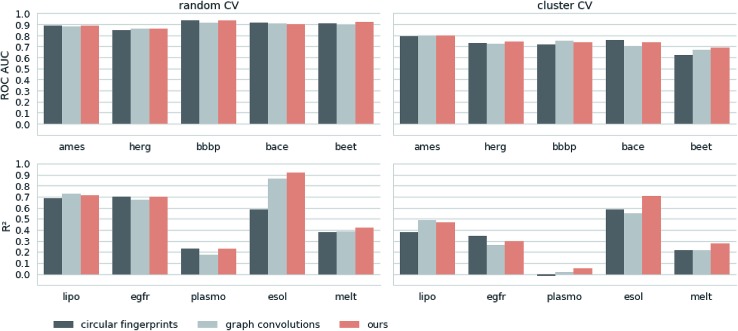
Results of the 5 regression and 5 classification QSAR-tasks. Separate results are shown for both cross-validation on random splits (random CV) and cross-validation on cluster splits (cluster CV). We compare the results of an SVM trained on our descriptors with the best model (SVM, RF and GB) trained on the best performing circular fingerprint as well as an end-to-end trained graph-convolution model after extensive hyperparameter optimization.

Next, we extracted molecular descriptors of the remaining (test) QSAR datasets (see Table 2) with the best performing translation model and benchmarked them as described in the Methods section. Fig. 3 shows the results of this evaluation for random-split and cluster-split cross-validation respectively, comparing our molecular descriptor to the best model based on the different circular fingerprints and the graph-convolution networks trained end-to-end for each QSAR dataset individually (see the ESI† for detailed results).

Generally, the hyperparameter-optimized methods perform on a comparable level for most of the QSAR tasks, each method showing at least on one task a slightly better mean performance over the different splits.

The lipophilicity and aqueous solubility datasets show the largest variance in performance between the models. The graph-convolution method outperforms the models based on the baseline fingerprint in predicting these physico-chemical endpoints. For the solubility endpoint, however, this is only true in the case of random splits. In the case of cluster splits, the graph-convolution model apparently fails to generalize on the hold-out clusters. This is probably due to the relatively small size of the solubility dataset. Since the graph-convolution method is trained end-to-end, it has to learn to extract meaningful features for each dataset from scratch which could lead to overfitting, if training data are limited. In contrast, the baseline fingerprints and our descriptors are built upon predefined or pretrained feature extraction methods respectively, independently from the task at hand. Our proposed molecular descriptors show good performance in predicting physico-chemical endpoints (lipophilicity, solubility and melting point) even in the cluster cross-validations on the small datasets (solubility and melting point).

Summing up, our proposed molecular descriptors exhibit competitive or better performance than the best baseline models in all investigated QSAR tasks.

Additionally, we would like to emphasize that we fixed our feature extraction method based on two datasets (Ames and lipophilicity on random splits) to avoid a model selection bias on the remaining test sets. This, however, was not done for the baseline methods. The fingerprint-based models could choose between nine different flavours of circular fingerprints and three different learning algorithms for each task respectively and due to the considerable training time the graph-convolution models were not trained in a nested cross-validation. Hence, it is remarkable that, although we applied a much harsher evaluation scheme on our method, it still achieved comparable – if not better – results to the baseline methods.

### 3.3 Virtual screening

The goal of ligand-based virtual screening (VS) is to rank a large set of compounds with respect to their activity on a certain target based on their similarity to some known active query compounds. It is based on the assumption that similar compounds will have a similar biological activity.

To investigate how well our descriptors are suited for ligand-based virtual screening, we followed the benchmark protocol of Riniker *et al.* to compare our extracted descriptors against other state-of-the-art molecular descriptors. In Table 3 the ranking performance of the descriptors is compared on the DUD and MUV databases respectively. On both databases our descriptor significantly outperformed the second best descriptor (*p* < 0.05). Thus, similarities measured between compounds in our proposed descriptor space are better correlated with their pharmacological similarity than similarities measured in the baseline fingerprint spaces.

**Table 3:** Results of the VS-experiment on the DUD and MUV databases for the best 10 descriptors respectively. *p*-Values of the Wilcoxon signed-ranked test between our descriptor and the second best are given respectively

(a) DUD: *p* = 5 × 10^–38^										
Descriptor	ours	laval	tt	lecfp4	lecfp6	ecfp4	rdk5	avalon	ecfp6	fcfp4
ROC-AUC	0.949	0.899	0.890	0.887	0.886	0.884	0.884	0.881	0.881	0.874

(b) MUV: *p* = 0.04										
Descriptor	ours	ap	tt	avalon	laval	ecfc4	rdk5	ecfc0	fcfc4	fcfp4
ROC-AUC	0.679	0.677	0.670	0.644	0.643	0.637	0.627	0.626	0.615	0.605

**Fig. 4:**
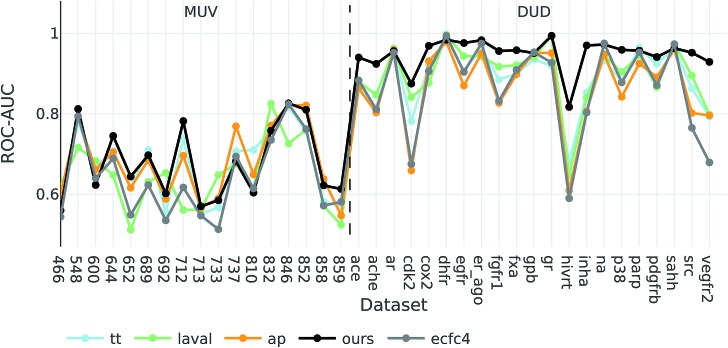
ROC-AUC of the VS experiments for each target for the overall best descriptors as well as ecfc4 fingerprints.

Interestingly, the best baseline descriptor in the DUD screen (laval) is only fifth in the MUV screen. The best baseline descriptor in the MUV screen (ap) is not even represented in the top ten performing descriptors in the DUD screen. Our descriptor, however, shows robust performance over all analysed targets (see Fig. 4), even though the translation model was selected based on its performance on two QSAR validation tasks.

### 3.4 Exploring the continuous descriptor space

As opposed to the previously discussed baseline fingerprints, our proposed descriptor is continuous and the encoding into the descriptor space is reversible, due to the decoder part of our translation model. This opens new possibilities in terms of compound optimization and exploration of the chemical space. As already shown by Gómez-Bombarelli *et al.*, a continuous encoding of a molecular structure enables us to explore the neighbourhood of this molecule by decoding from points close to the query molecule's embedding.

**Fig. 5:**
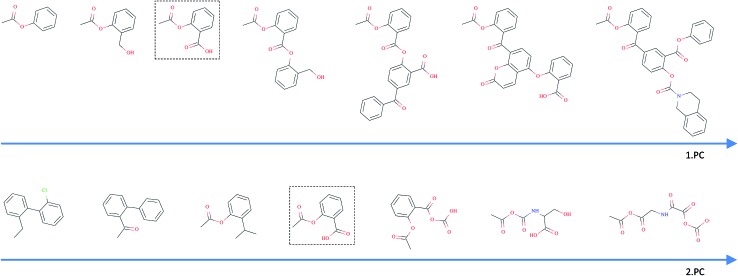
Shifting of an example query molecule (here acetylsalicylic acid) along the first (top) and second (bottom) principal components of the pretraining dataset. The query molecule (dashed box) was encoded in our descriptor space, which was iteratively shifted – in both negative and positive directions – and decoded back to a SMILES.

In Fig. 5, we incrementally shift the embedding of a query molecule in two different directions and decode it back to a molecule. The directions we are shifting the molecule's embedding along are defined by the first and second principal component of the pretraining dataset (molecules from PubChem and ZINC) in our descriptor space. We observe that the incremental shifts in the continuous descriptor space correspond to smooth transitions in the discrete chemical space. Apparently, the first principal component of our pretraining dataset correlates with the size of molecules: adding or subtracting a value along this axis corresponds to adding or removing atoms from the structure. Shifts along the second principal component of the pretraining dataset seem to be correlated with altering the molecule's polarity. To objectively analyse potential correlations between certain axes in the continuous descriptor space and molecular properties, we repeated the experiment with 1000 randomly picked compounds from the validation dataset and shifted each of them 10 steps in the negative and 10 steps in the positive direction along the two principle components, respectively. The mean Spearman correlation coefficient *r* between the compound's molar weight and the respective step along the first principle component was *r* = 0.9470 (*p* = 0.00048). The mean correlation between the compound's partition coefficient log *P* and the respective step along the second principle component was *r* = –0.916 (*p* = 0.00015). These results suggest a general correlation between shifts in certain directions in the descriptor space and certain molecular properties.

All analysed points along these two axes, when decoded, resulted in a valid SMILES (interpretable by RDKit). To further investigate how well our model is suited to explore around arbitrary molecule representations in arbitrary directions, we iteratively moved along 100 random directions for 1000 randomly picked compounds, respectively (see the ESI† for examples of generated compounds). Table 4 shows the aggregated results for this exploration. As expected, we observed a clear correlation between the (Euclidean) distance in our descriptor space and the (Tanimoto) distance in the circular fingerprint space. Thus, shifting the representation of a molecule in our descriptor space corresponds to gradual transitions in the chemical space. On average, even if shifted over long distances, our model succeeds in generating a high proportion of valid SMILES (>97%). If the most probable output of the model's beam search decoder results in an invalid SMILES, we observe that it is likely that one of the next most probable sequences results in a valid SMILES (>99%).

**Table 4:** Aggregated results of the exploration of our descriptor space in 100 different random directions for 1000 different compounds in successive steps. For each step, *d*_Euclidean_ is the mean Euclidean distance between the representations at this step and the representation of the respective starting compounds. *d*_Tanimoto_ is the mean Tanimoto distance between the ecfc4 fingerprints of the successfully decoded compounds and their starting compounds at each step. Rate_1_, rate_2_, and rate_3_ describe the mean valid SMILES reconstruction rate, taking the first one, two and three most probable beam search outputs into account respectively

*d*_Euclidean_	2.0	3.9	5.7	7.5	9.2	10.7	12.2	13.5	14.8	15.9	16.9	17.7
*d*_Tanimoto_	0.02	0.02	0.05	0.10	0.19	0.31	0.46	0.60	0.71	0.78	0.83	0.86
Rate_1_	1.00	1.00	1.00	1.00	0.99	0.99	0.98	0.98	0.98	0.97	0.97	0.97
Rate_2_	1.00	1.00	1.00	1.00	1.00	1.00	0.99	0.99	0.99	0.99	0.99	0.99
Rate_3_	1.00	1.00	1.00	1.00	1.00	1.00	1.00	1.00	1.00	1.00	1.00	0.99

In a similar study Blaschke *et al.*, for example, analyzed 4 different autoencoder frameworks on the SMILES to SMILES reconstruction task and reported a valid SMILES proportion of only approximately 20% using their best model, if moved away by a similar (Tanimoto) distance (note, however, that a direct comparison is problematic since Blaschke *et al.* sampled directly from the probability distribution of the last decoder layer and did not perform a beam search as we did).^51^ Another study by Segler *et al.* demonstrates that a simple RNN solely trained on generating SMILES sequences (no encoder/decoder framework) can obtain similar high valid SMILES ratios of 96% with random sampling.^52^

## 4 Conclusion

We proposed a novel methodology that is able to learn to extract meaningful molecular descriptors, solely by an unsupervised training on a large dataset of molecular structures. We showed that the molecular descriptors extracted by our method significantly outperform state-of-the-art molecular fingerprints in ligand-based virtual screening (VS) experiments. Moreover, we show that machine learning models based on our descriptor perform similarly – if not better – on various quantitative structure–activity relationships (QSAR tasks), when compared to multiple state-of-the-art molecular fingerprints and computationally expensive graph-convolution models. Generally, our proposed descriptors show, compared to the baseline methods, consistent performance in all experiments, even across different experimental concepts such as QSAR and VS. We believe that our method combines the advantages of both baseline models. Our method does not depend on fixed feature extraction rules but learns its own extraction method in a data-driven way. However, since it is pretrained on a large set of molecules, the resulting features generalize well and are less prone to overfitting.

As we focused in this work on translating between different string-based molecular representations, an evident follow-up would be the translation of conceptually different molecular representations such as the molecular graph or 3D-structure-based representations like the van der Waals and/or electronegative potential surface.

Since our proposed molecular descriptors are continuous and can be translated back into a valid molecular structure, they open new possibilities in terms of compound optimization and navigation of the chemical space. We observe smooth and meaningful transitions in the chemical structure when a molecule's embedding is shifted in certain directions, where shifts along different axes in our descriptor space correspond to different structural and functional properties in the chemical space.

Moreover, Gómez-Bombarelli *et al.* already showed that their autoencoder framework could be utilized to automatically design molecules with respect to multiple properties such as synthetic accessibility and drug-likeness. Since our model's latent space was shown to be significantly better correlated with the molecule's biochemical properties, we think that our proposed translation method could significantly improve such a method's ability to generate and optimize molecules, also enabling optimization with respect to biological activity. These aspects will be explored and discussed in an upcoming study.

## Availability

The source code and a pretrained model (to extract our proposed molecular descriptors out of the box) are available at https://github.com/jrwnter/cddd.

## Conflicts of interest

There are no conflicts to declare.

## Supplementary Material

Supplementary information
