# Supplementary material for: Learning continuous and data-driven molecular descriptors by translating equivalent chemical representations
Source: Chem Sci. 2018 Nov 19;10(6):1692–701. doi: 10.1039/c8sc04175j (PMC6368215; doi:10.1039/c8sc04175j)
Supplement: Supplementary file 1 [file SC-010-C8SC04175J-s001.pdf]

Electronic Supplementary Material (ESI) for Chemical Science.
This journal is © The Royal Society of Chemistry 2018

Supporting Information for Learning Continuous and Data-Driven Molecular Descriptors by Translating Equivalent Chemical Representations

Robin Winter,[*,†,‡] Floriane Montanari,[†] Frank Noé,[‡] and Djork-Arné Clevert[*,†]

†*Department of Bioinformatics, Bayer AG, 13353 Berlin, Germany*

‡*Department of Mathematics and Computer Science, Freie Universität Berlin, 14195 Berlin, Germany*

E-mail: robin.winter@bayer.com; djork-arne.clevert@bayer.com

Common neural network architectures

In the following we introduce the basic concepts of the different neural network architectures used in our translation model

Fully-connected Neural Network

The most basic form of a Deep Neural Network is the Fully-connected Neural Network (FNN). In an FNN, each neuron in a layer of the network is connected to each neuron in the previous layer (see Figure 1 a). Thus, the output of a neuron in a fully-connected layer is a linear combination of all outputs of the previous layer, usually followed by a non-linear function.

Convolutional Neural Network

A convolutional neural network (CNN) builds up on multiple small kernels which are convolved with the outputs of the previous layer. In contrast to a neuron in a fully-connected layer, each output of a convolutional-layer is only a linear combination of neighboring outputs in the previous layer (see Figure 1 b). The number of considered neighbors is defined by the size of the kernel. Since the same kernels are applied along all outputs of the previous layer, a convolutional architecture is especially well suited for recognition of patterns in the input, independently of their exact position. Hence, CNNs are popular in image analysis tasks but were also successfully applied on sequence-based data.[1,2]

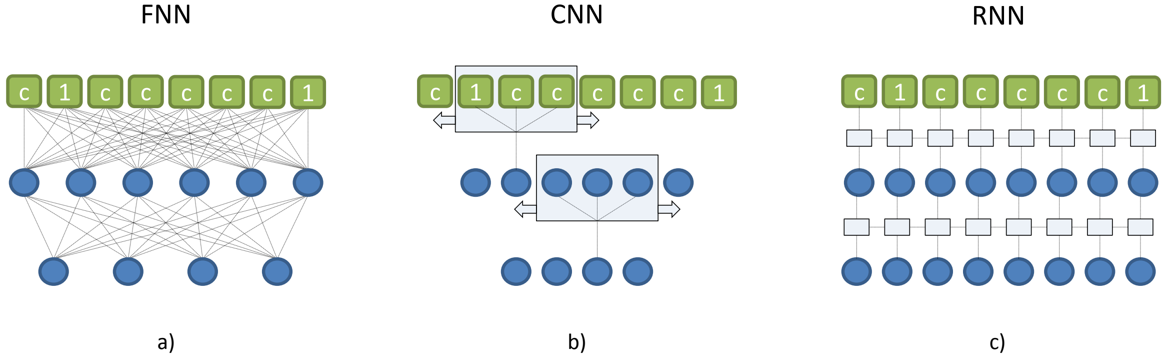

Figure 1: Three different 2-layer neural network architectures with SMILES representation of benzene as input. a) Fully-connected Neural Network (FNN): Each neuron is connected with each neuron/input of the previous layer. b) Convolutional Neural Network (CNN): The Input to a certain layer is convolved with a kernel of size 3. c) Recurrent Neural Network (RNN): Illustration of a RNN with an unrolled graph. Note that a 2-layer of RNN has two separate cell states (blue boxes)

Recurrent Neural Network

A more tailored architecture for sequence-based data is the Recurrent Neural Network (RNN). In contrast to FNN and CNN where all information flows in one direction, from the input layer through the hidden layers to the output layer (feed-forward neural networks), an RNN

has an additional feedback loop (recurrence) through an internal memory state. An RNN processes a sequence step by step while updating its memory state concurrently. Hence, the activation of a neuron at step t is not only dependent on the input at t but also on the state at position $t-1$. By including a memory cell, an RNN is in theory able to model long-term dependencies in sequential data, such as keeping track of opening and closing brackets in the SMILES syntax. The concept of a neural network with a feedback loop can be simplified by unrolling the RNN network over the whole input sequence (see Figure 1 c). Unrolling the graph emphasizes that an RNN which takes long sequences as input becomes a very deep neural network suffering from problems such as vanishing or exploding gradients[3]. To avoid this problem, Hochreiter et al. proposed the *Long short-term memory* (LSTM) network, which extends the RNN architecture by an *input gate*, an *output gate* and a *forget gate*.[4] In this work we use a modified version of the LSTM: the *gated recurrent unit* (GRU).[5]

Baseline Molecular Descriptors

In this section we will introduce the basic concepts of the different molecular descriptors we used as baseline.

Circular fingerprints like the extended-connectivity fingerprints (ECFPs) were introduced for the purpose of building machine learning models for quantitative structure-activity relationships (QSAR) models. This class of fingerprints iterates over the non hydrogen atoms of a molecular graph and encodes the neighbourhood up to a given radius using a linear hash function. The resulting set of neighbourhood hash codes for a dataset can then be handled as a sparse matrix or folded to a much smaller size (1024 or 2048 are typical folding size choices). Folding such a potentially large bit space (2^{32}) to a much smaller space produces collisions in the final fingerprint vector, where two different neighbourhood codes could end up at the same position in the folded vector, resulting in a loss of information. Additionally, once folded, it is impossible to trace back important bits to actual compound substructures,

and therefore the model interpretability is lost.

In the ligand-based virtual screening experiments we followed the benchmarking protocol proposed by Riniker et al.[6] In this protocol we benchmarked our proposed descriptor against the 14 fingerprints available in the pipeline. Accordingly Rinker et al., these fingerprints can be divided into four different classes: dictionary-based (e.g. Molecular ACCess System MACCS), topological or path-based (e.g. atom pair (AP) fingerprints or topological torsions (TT)), circular fingerprints (e.g. ECFP) and pharmacophores (eg. FCFP). For a more comprehensive description of the different baseline fingerprints we refer to the work of Rinker et al..

QSAR modelling

We used the Python library RDKit (v.2017.09.2.0) to calculated Morgan fingerprints with radius 1, 2 and 3 (equivalent to ecfc2, ecfc4, ecfc6) each folded to 512, 1024 and 2048 bits, resulting in nine different baseline molecular descriptors.

Three different machine learning algorithms were used to model the QSAR tasks: Random Forest (RF), Support Vector Machine (SVM) and Gradient Boosting (GB). We utilized the Python library sklearn (v.0.19.1) to build, train and cross-validate the models. The hyperparameter optimization was performed for each QSAR task, model and fingerprint triplet individually. The hyperparameter grid for the different models was defined as follows:

- Random Forest:
  - Number of estimators (trees): 20, 100 and 200
- Support Vector Machine:
  - Penalty parameter C of the error term: 0.1, 0.5, 1 , 3 5, 10, 30 and 50
  - Coefficient gamma for the RBF-kernel: $1/(N_f + b)$, where N_f is the number of features and b is picked from -300, -150, -50, -15, 0, 15, 50, 150 and 300

- Gradient Boosting:
  - Number of estimators: 20, 100 and 200
  - Learning rate: 0.5, 0.1 and 0.01

In addition to the classical machine learning methods trained on circular fingerprints we also trained graph-convolution models on the different QSAR tasks. Briefly, the architecture consists of two successive graph convolution layers, an input atom vector of size 75 and rectified linear units (ReLU) were used as non linearity. For each task a individual graph-convolution model were trained and optimized with respect to following hyperparameters:

- batch size: 64, 128 and 256
- learning rate: 0.0001, 0.0005, 0.001 and 0.005
- convolutional filters: 64, 128 and 256
- dimension of the dense layer: 128, 256, 512, 1024, and 2048
- number of epochs: 40, 60 and 100

Translation Model Architecture

As final translation model we selected the model with the best performance on the validation QSAR tasks (see Figure 2). For the encoding part we stacked 3 GRU cells with 512, 1024 and 2048 units respectively. The state of each GRU cell is concatenated and fed into a fully-connected layer with 512 neurons and hyperbolic tangent activation function. The output of this layer (values between -1 and 1) is the latent space which can be used as molecular descriptor in the inference time. The decoding part takes this latent space as input and feeds it into a fully-connected layer with 512 + 1024 + 2048 = 3584 neurons. This output is split into 3 parts and used to initialize 3 stacked GRU cells with 512, 1024 and 2048 units respectively. The output of the GRU cells is mapped to predicted probabilities for the

different tokens via a fully-connected layer.

The classifier network consists of a stack of three fully-connected layers with 512, 128 and 9 neurons respectively, mapping the latent space to the molecular property vector. The model was trained on translating between SMILES and canonical SMILES representations. Both sequences were tokenized as described in the method section and fed into the network.

In order to make the model more robust to unseen data, input dropout was applied on a character level (15%) and noise sampled from a zero-centered normal distribution with a standard derivation of 0.05. We used an Adam optimizer[7] with a learning rate of $5 * 10^{-4}$ which was decreased by a factor of 0.9 every 50000 steps. The batch size was set to 64. To handle input sequences of different length we used a so-called bucketing approach. This means that we sort the sequences by their length in different buckets (in our case 10) and only feed sequences from the same bucket in each step. All sequences were padded to longest sequence in each bucket. We used the framework TensorFlow 1.4.1[8] to build and execute our proposed model.

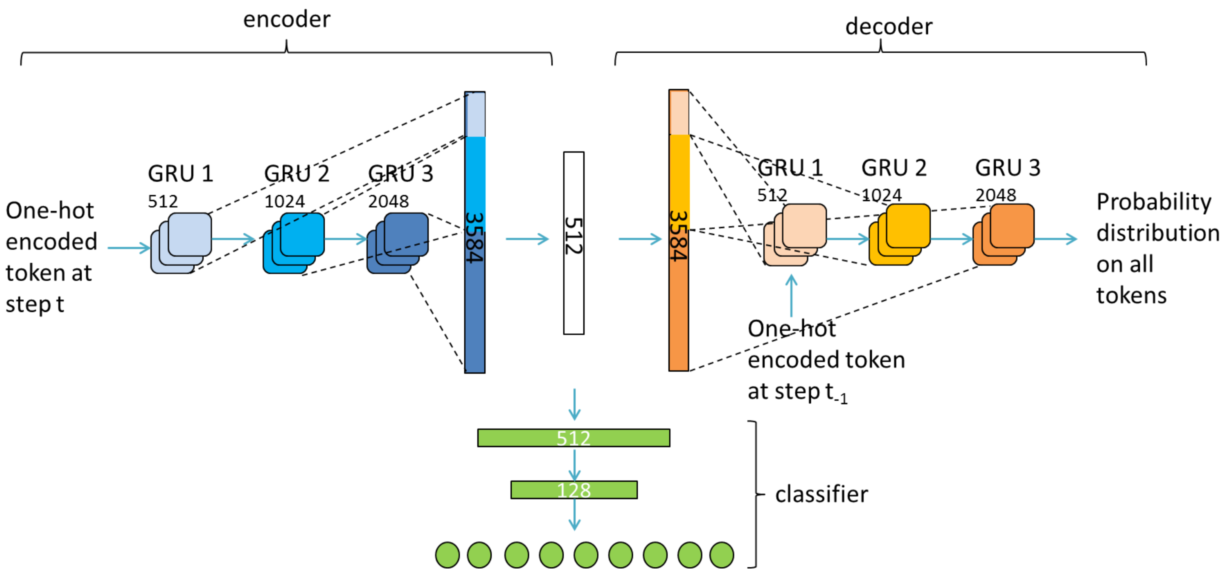

Figure 2: Final model architecture.

QSAR results

Tables 1, 2, 3 and 4 show the detailed results for the hyperparameter optimized models. For the baseline fingerprint models, only the result for the best performing fingerprint is shown. For each task we show the performance of an SVM on our descriptor (ours), the performance of a graph-convolution model (GC) and the best performing model-fingerprint combination (RF, SVM or GB). For the classification tasks we measure the area under the Receiver Operating Characteristic curve (roc-auc), the area under the precision-recall curve (pr-auc) accuracy (acc), F1-measure (f1). For the regression tasks we measure the coefficient of determination (r^2), Spearman's rank correlation coefficient (r), mean squared error (mse) and mean absolute error (mae).

Table 1: Averaged results for the four classification QSAR task in random-split cross-validation after hyperparameter optimization.

Task	roc-auc	pr-auc	acc	f1	Descriptor	Model
ames	0.89	0.91	0.81	0.83	ecfc2 1024	RF
	0.88	0.90	0.81	0.83	-	GC
	0.89	0.91	0.82	0.83	ours	SVM
herg	0.85	0.94	0.82	0.89	ecfc4 1024	RF
	0.86	0.94	0.83	0.89	-	GC
	0.86	0.94	0.82	0.88	ours	SVM
bbbp	0.93	0.97	0.90	0.94	ecfc2 512	RF
	0.92	0.97	0.88	0.92	-	GC
	0.93	0.97	0.90	0.94	ours	SVM
bace	0.91	0.89	0.84	0.82	ecfc2 512	RF
	0.91	0.88	0.82	0.81	-	GC
	0.90	0.86	0.84	0.83	ours	SVM
beetox	0.91	0.88	0.89	0.69	ecfc6 2048	RF
	0.89	0.79	0.88	0.69	-	GC
	0.92	0.83	0.92	0.80	ours	SVM

# VS Results

Tables 5 and 6 show the detailed results for the virtual screening experiments performed on the DUD and MUV databases. Our descriptor (ours) is compared to different baseline

Table 2: Averaged results for the four regression QSAR task in random-split cross-validation after hyperparameter optimization.

Task	r^2	r	mse	mae	Descriptor	Model
lipo	0.69	0.83	0.42	0.46	ecfc2 1024	SVM
	0.73	0.84	0.36	0.44	-	GC
	0.72	0.83	0.38	0.46	ours	SVM
egfr	0.70	0.84	0.62	0.57	ecfc4 2048	RF
	0.67	0.83	0.68	0.60	-	GC
	0.70	0.85	0.62	0.57	ours	SVM
plasmo	0.23	0.45	0.25	0.38	ecfc2 2048	SVM
	0.18	0.41	0.27	0.40	-	GC
	0.23	0.45	0.25	0.38	ours	SVM
esol	0.58	0.82	1.38	0.91	ecfc6 1024	SVM
	0.86	0.92	0.58	0.56	-	GC
	0.92	0.96	0.34	0.42	ours	SVM
melt	0.38	0.62	1700	33	ecfc2 2048	SVM
	0.39	0.67	1700	34	-	GC
	0.42	0.64	1600	32	ours	SVM

Table 3: Averaged results for the four classification QSAR task in cluster-split cross-validation after hyperparameter optimization.

Task	roc-auc	pr-auc	acc	f1	Descriptor	Model
ames	0.79	0.81	0.74	0.70	ecfc4 2048	SVM
	0.80	0.80	0.74	0.74	-	GC
	0.80	0.82	0.74	0.71	ours	SVM
herg	0.73	0.89	0.76	0.85	ecfc4 2048	RF
	0.72	0.89	0.77	0.86	-	GC
	0.75	0.90	0.76	0.84	ours	SVM
bbbp	0.72	0.88	0.79	0.84	ecfc4 2048	RF
	0.75	0.90	0.77	0.83	-	GC
	0.74	0.88	0.81	0.86	ours	SVM
bace	0.76	0.70	0.67	0.59	ecfc2 2048	GB
	0.70	0.67	0.55	0.52	-	GC
	0.74	0.67	0.65	0.55	ours	SVM
beetox	0.62	0.39	0.75	0.00	ecfc6 512	GB
	0.67	0.48	0.72	0.20	-	GC
	0.69	0.45	0.78	0.21	ours	SVM

Table 4: Averaged results for the four regression QSAR task in cluster-split cross-validation after hyperparameter optimization.

Task	r^2	r	mse	mae	Descriptor	Model
lipo	0.38	0.70	0.84	0.68	ecfc4 2048	RF
	0.49	0.69	0.68	0.64	-	GC
	0.47	0.69	0.71	0.65	ours	SVM
egfr	0.34	0.58	1.01	0.77	ecfc6 1024	RF
	0.26	0.51	1.16	0.86	-	GC
	0.30	0.55	1.09	0.81	ours	SVM
plasmo	-0.02	0.21	0.33	0.44	ecfc6 1024	RF
	0.02	0.20	0.32	0.44	-	GC
	0.05	0.24	0.31	0.43	ours	SVM
esol	0.58	0.82	1.38	0.91	ecfc6 2048	SVM
	0.55	0.76	1.62	0.96	-	GC
	0.70	0.89	1.01	0.75	ours	SVM
melt	0.22	0.51	1800	34	ecfc2 1024	SVM
	0.21	0.48	1930	34	-	GC
	0.28	0.55	1700	32	ours	SVM

descriptors by the are under the Receiver Operating Characteristic curve (ROC-AUC), the Enrichment Factor (EF) for the top 5% ranked compounds 5%, the Robust Initial Enhancement (RIE) with parameter $\alpha = 20$ and the Boltzmann-Enhanced Discrimination of ROC (BEDROC) with parameter $\alpha = 20$. For detailed description of the baseline descriptors and evaluation metrics, we refer the reader to the work of Riniker et al.[6]

Table 5: Results of the ligand-based virtual screen of the DUD database.

Descriptor	ROC-AUC	BEDROC	RIE	EF5
ours	**0.949**	**0.792**	**12.485**	**15.294**
laval	0.899	0.746	11.750	14.216
tt	0.890	0.744	11.718	14.257
lecfp4	0.887	0.771	12.138	14.813
lecfp6	0.886	0.767	12.072	14.691
ecfp4	0.884	0.764	12.024	14.623
rdk5	0.884	0.747	11.761	14.291
avalon	0.881	0.733	11.537	13.922
ecfp6	0.881	0.755	11.879	14.429
fcfp4	0.874	0.754	11.868	14.472
ap	0.868	0.717	11.298	13.676
ecfc4	0.867	0.749	11.782	14.354
maccs	0.863	0.667	10.511	12.730
fcfc4	0.852	0.728	11.459	13.891
ecfc0	0.805	0.570	8.969	10.634

Table 6: Results of the ligand-based virtual screen of the MUV database.

Descriptor	ROC-AUC	BEDROC20	RIE20	EF5
ours	**0.679**	**0.195**	**3.826**	**4.258**
ap	0.677	0.176	3.440	3.737
tt	0.670	0.191	3.746	4.066
avalon	0.644	0.174	3.403	3.661
laval	0.643	0.172	3.375	3.659
ecfc4	0.637	0.181	3.540	3.895
rdk5	0.627	0.177	3.473	3.747
ecfc0	0.626	0.130	2.541	2.816
fcfc4	0.615	0.163	3.184	3.471
fcfp4	0.605	0.175	3.420	3.706
lecfp4	0.601	0.178	3.480	3.774
lecfp6	0.599	0.178	3.481	3.768
ecfp4	0.599	0.176	3.447	3.753
ecfp6	0.591	0.175	3.433	3.776
maccs	0.578	0.127	2.482	2.705

Timing

Another point to consider, when comparing our proposed method with the baseline, is the time needed to extract the molecular descriptors and train the machine learning algorithm. Calculating Morgan fingerprints with RDKit takes on a single CPU only a few seconds for a dataset of 10.000 compounds. If ran on a modern GPU, our proposed model takes approximately the same time. On a single CPU core, however, this computational time increases approximately by a factor of 100. Training an RF for this size of dataset on our descriptors is in the order of seconds. An SVM, that scales with number of input features, takes, in the order of minutes for the best baseline fingerprints (ecfpc4 2048), and in the order of seconds for our descriptors (512 dimensional). The best performing graph-convolution model, on the other side, takes approximately 30 minutes to train on a modern GPU (on a single CPU this method would not be computational feasible). Thus, with a GPU at hand, running a QSAR experiment with our proposed descriptors is on the same timescale as with state-of-the-art molecular fingerprints, while being significantly faster than training a graph-convolution model. One way to make our encoder faster would be to replace the GRU cells by one-dimensional convolutional layers. In our experiments however these do not perform as well in the downstream QSAR validation sets.

Generated compounds

Figure 3 and 4 depicts examples of compounds generated for the experiment in section 3.4 in the main article. The compounds shown are randomly picked and had a (ecfp4-) tanimoto distance greater than 0.5 to the starting compound.

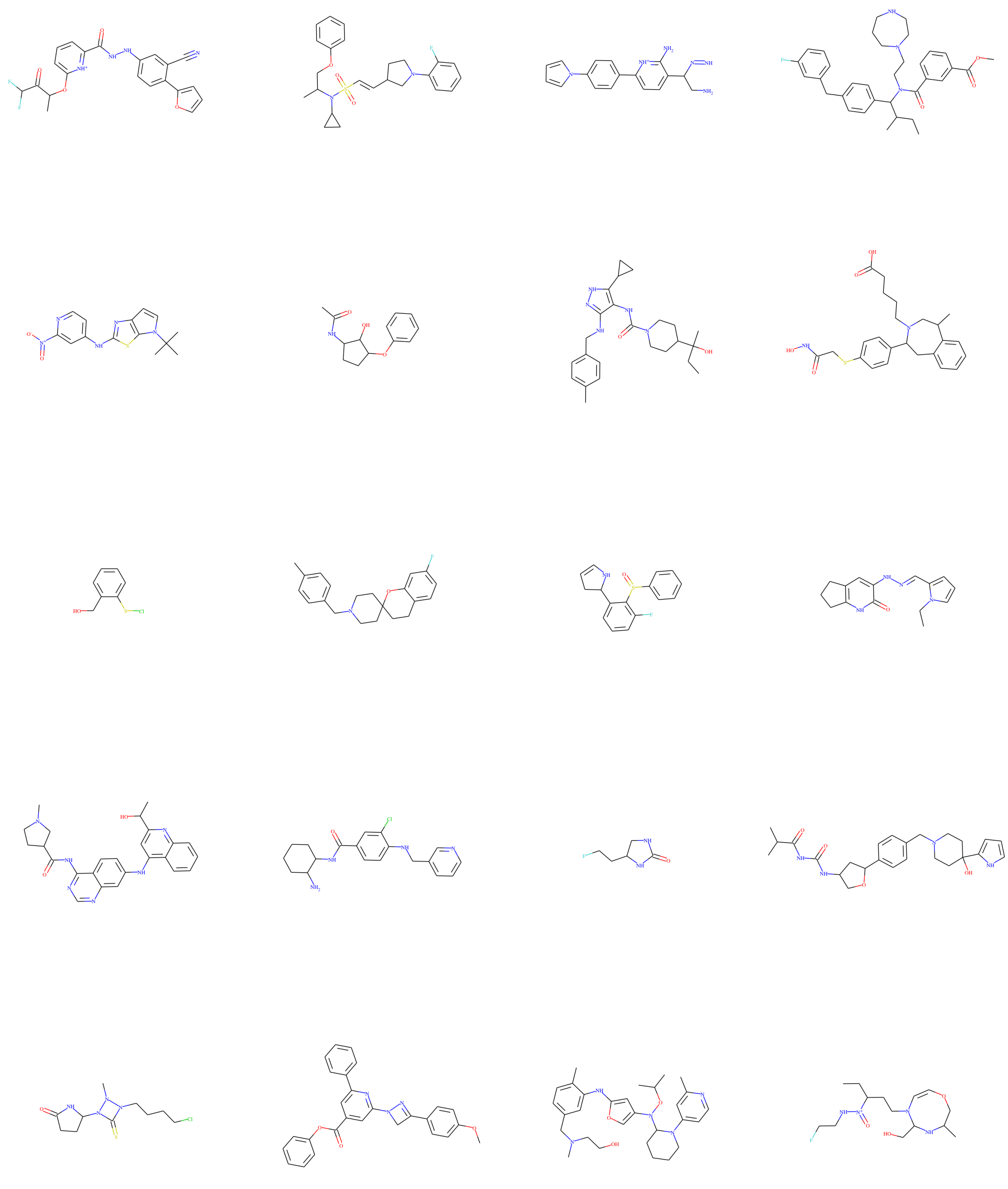

Figure 3: Examples of generated compounds.

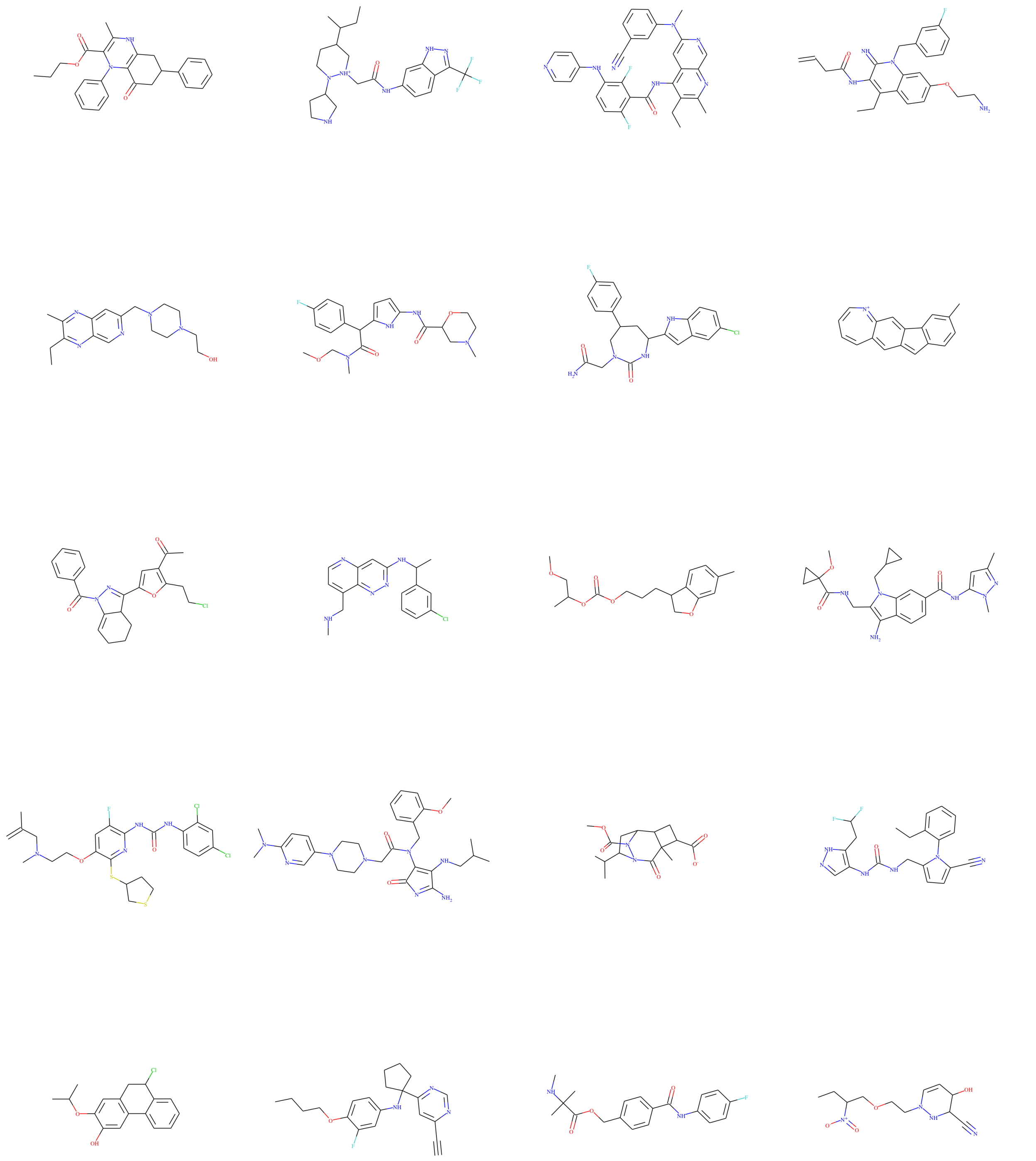

Figure 4: Examples of generated compounds.

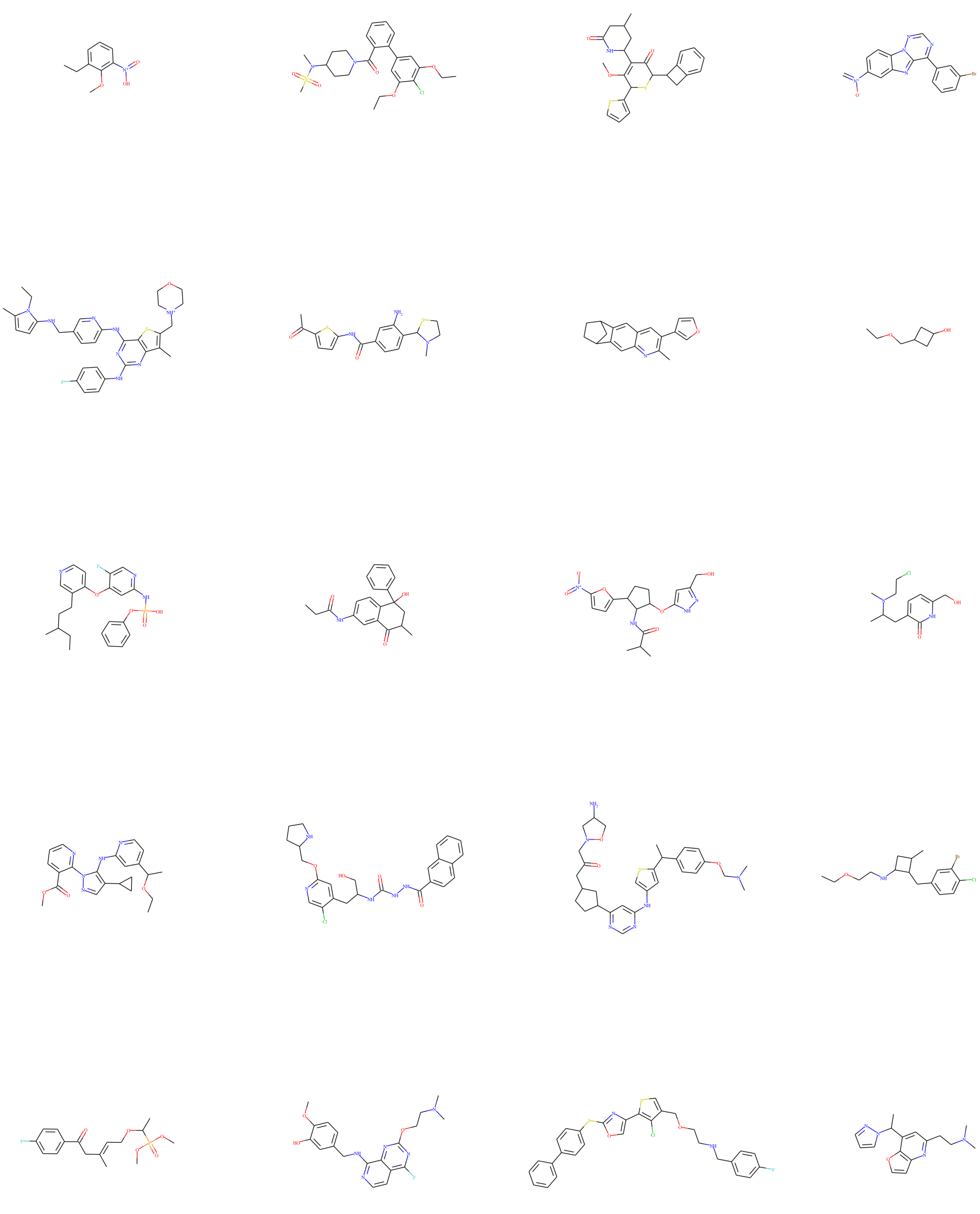

Figure 5: Examples of generated compounds.

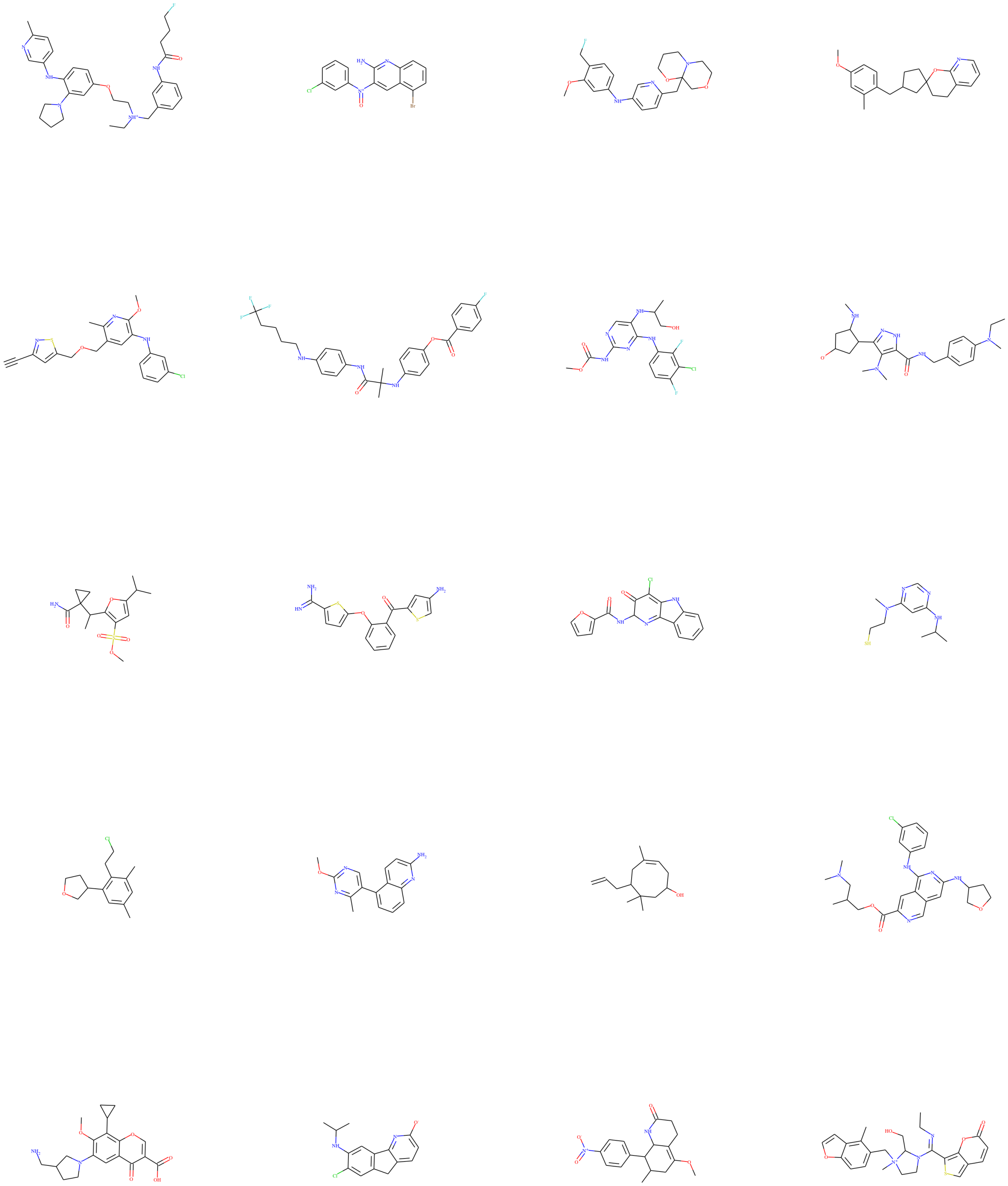

Figure 6: Examples of generated compounds.

References

(1) Krizhevsky, A.; Sutskever, I.; Hinton, G. E. Imagenet classification with deep convolutional neural networks. Advances in neural information processing systems. 2012; pp 1097–1105.

(2) Gehring, J.; Auli, M.; Grangier, D.; Yarats, D.; Dauphin, Y. N. Convolutional sequence to sequence learning. *arXiv preprint arXiv:1705.03122* **2017**,

(3) Hochreiter, S.; Bengio, Y.; Frasconi, P.; Schmidhuber, J. Gradient flow in recurrent nets: the difficulty of learning long-term dependencies. 2001.

(4) Hochreiter, S.; Schmidhuber, J. Long short-term memory. *Neural computation* **1997**, *9*, 1735–1780.

(5) Chung, J.; Gulcehre, C.; Cho, K.; Bengio, Y. Empirical evaluation of gated recurrent neural networks on sequence modeling. *arXiv preprint arXiv:1412.3555* **2014**,

(6) Riniker, S.; Landrum, G. A. Open-source platform to benchmark fingerprints for ligand-based virtual screening. *J. Cheminf.* **2013**, *5*, 26.

(7) Kingma, D. P.; Ba, J. Adam: A method for stochastic optimization. *arXiv preprint arXiv:1412.6980* **2014**,

(8) Abadi, M. et al. TensorFlow: Large-Scale Machine Learning on Heterogeneous Systems. 2015; `https://www.tensorflow.org/`, Software available from tensorflow.org.
